# In-Line Observation of Laser Cladding Processes via Atomic Emission Spectroscopy

**DOI:** 10.3390/ma14164401

**Published:** 2021-08-06

**Authors:** Malte Schmidt, Philipp Huke, Christoph Gerhard, Knut Partes

**Affiliations:** 1 Department of Engineering Sciences, Jade University of Applied Sciences, Friedrich-Paffrath-Str. 101, 26389 Wilhelmshaven, Germany; malte.schmidt@jade-hs.de; 2 Institute for Laser and Optics (ILO), University of Applied Sciences Emden/Leer, Constantiaplatz 4, 26723 Emden, Germany; philipp.huke@hs-emden-leer.de; 3 Faculty of Engineering and Health, University of Applied Sciences and Arts, Von-Ossietzky-Straße 99, 37085 Göttingen, Germany; christoph.gerhard@hawk.de

**Keywords:** optical emission spectroscopy (OES), direct metal deposition (DMD), laser cladding

## Abstract

Direct metal deposition (DMD) can be used for the cladding of surfaces as well as repairing and additive manufacturing of parts and features. Process monitoring and control methods ensure a consistent quality during manufacturing. Monitoring by optical emission spectroscopy of the process radiation can provide information on process conditions and the deposition layer. The object of this work is to measure optical emissions from the process using a spectrometer and identify element lines within the spectra. Single spectra have been recorded from the process. Single tracks of Co-based powder (MetcoClad21) were clad on an S235 base material. The influence of varying process parameters on the incidence and intensity of element lines has been investigated. Moreover, the interactions between the laser beam, powder jet, and substrate with regard to spectral emissions have been examined individually. The results showed that element lines do not occur regularly. Therefore, single spectra are sorted into spectra including element lines (type A) and those not including element lines (type B). Furthermore, only non-ionised elements could be detected, with chromium appearing frequently. It was shown that increasing the laser power increases the incidence of type A spectra and the intensity of specific Cr I lines. Moreover, element lines only occurred frequently during the interaction of the laser beam with the melt pool of the deposition layer.

## 1. Introduction

In multiple industry sectors, direct metal deposition (DMD) is present today. It can provide a bandwidth of different processes such as cladding of surfaces [1,2] as well as the repairing [3,4] and additive manufacturing of parts [5,6]. In this process, a high-power laser generates a melt pool on the surface of a metallic material, and filler material (commonly in powder form) is simultaneously delivered into the melt pool. By controlling the material flow and the laser power, functional layers can be placed on surfaces by putting tracks side by side. This process can be used for several types of applications, e.g., cladding of surfaces for wear protection purposes, repairing large-scale industrial components, and additive manufacturing of parts and features. Due to the large number of available powder materials, the process can be used quite flexibly [7]. In order to achieve consistent quality during manufacturing, numerous process monitoring and control methods have been developed [8,9,10,11,12,13,14].

A specific field of process monitoring methods includes the analysis of optical emission that is provided by the process itself. Throughout multiple laser welding processes, optical emission spectroscopy is used. Investigations on laser lap welding used the intensity of specific spectral lines to determine the electron temperature within the plasma plume and detect defects [15,16,17]. Moreover, the detection of defects by process radiation emissions was theoretically analysed and discussed [18]. In situ chemical composition monitoring was performed by Huber during deep penetration laser welding. The procedure is partly based on the approach of laser-induced breakdown spectroscopy (LIBS). Here, spectral lines are measured and identified using an atomic spectra database, and element contents are related to the intensity of one or several element spectral lines [19]. The intensity of spectral lines can also be used as an indicator for homogeneity within a melt pool during laser-based experimental materials development where portions of material are remelted through an oscillating laser beam. In this specific case, the intensity of Cr spectral lines, provided by process emissions, can be used as a suitable measurement of blending within the microstructure [20]. 

Optical emission spectroscopy (OES) has been performed in DMD processes, too. In [21], a method for lack-of-fusion defect detections in titanium alloy layers has been investigated. Data of multiple sensors were combined with X-ray computed tomography data using machine-learning algorithms. It has been investigated that the laser power, powder flow rate, and hatch pattern have a statistically significant effect on the length of pores. The sensor system is capable of predicting lack-of-fusion defects across a layer. Several investigations on defect detection have been done [22,23]. Using the spectroscopic detection of specific spectral lines allows for generating different information about the manufactured part and the process parameters. For instance, the detection of Cr spectral lines in the course of DMD processes has been investigated by Bartkowiak. Here, it was confirmed that emission spectroscopy can be used as an in situ monitoring system with lower laser powers [24]. The loss of chromium during the direct metal deposition of the Ni-based alloy 718 was investigated by Kisielewicz. Optical emission data were measured from the plasma plume above the melt pool with increasing laser power. Multiple Cr I spectral lines have been identified. Specimens, built with higher laser power, showed a lower Cr content related to those built with lower laser power. The intensity of Cr lines increases with higher laser power. Higher energy input led to increasing vaporisation and the depletion of Cr atoms from the melt pool [25]. This observation exemplifies the importance of in situ monitoring of laser welding processes in order to address the formation of non-stoichiometric laser-generated material.

Regarding such monitoring of the chemical composition during deep penetration laser welding [19], the methodology has also been investigated in DMD processes. Studies on real-time Cr measurement were performed by using spectral line intensity ratios of neutral Cr and Fe lines. The element contents are determined on the basis of calibration curves. Approaches for element content prediction using the detection of plasma temperature or electron density occurred to be less accurate [26]. The monitoring of nickel and chromium content had also been performed using line ratios of specific neutral Ni and Cr lines. The prediction calibration curve was built through multiple experiments using different powder materials with varying Ni content [27]. In order to expand the quantity of elements to be measured, Wang and Liu used four different Ni-based alloy powders on an Fe-based substrate. Calibration curves for different elements were generated by correlating the weight ratio and the line intensity ratio of two elements. The prediction showed quite suitable results, although elements with lower concentration within the alloy could be measured with a much lower accuracy [28]. In [29], four composition monitoring methods are compared to predict the Al content within an Al-Ti-powder deposited layer. The investigation focuses on the accuracy and stability of element concentration prediction of each method. Input data for every method contain the line-intensity ratio of both Al and Ti lines. Additionally, the integrated intensity of a specific bandwidth is included. The methods to be compared are calibration curves, artificial neural network (ANN), partial least square regression (PLSR), and support vector regression (SVR). It has been observed that SVR using line intensity and integrated intensity data provides an improved performance for predicting the Al concentration compared to the other three methods.

Different approaches for chemical composition monitoring during DMD are based on the integration of separate LIBS systems. For instance, a LIBS probe was attached to a DMD processing head and aligned to measure the chemical composition within the powder jet. Compared to measurements at solid metal targets, a poor reproducibility within the powder jet was observed. This effect is explained by the low probability of a reproduceable interaction between moving powder particles and the laser beam [30]. In contrast, the use of LIBS on already applied coatings was investigated. Element spectral lines were used to determine the degree of mixing. The LIBS probe was attached to the processing head and performed measurements shortly after deposition. Results confirmed the method being suitable for quality assurance during the laser cladding process [31]. Against this background, the impact of process parameters during laser cladding on emission spectra formed in the course of the process was investigated in the present work.

## 2. Materials and Methods

**Figure 1:**
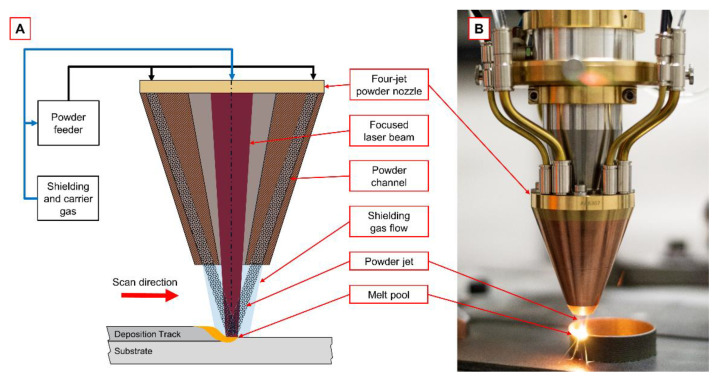
(**A**) Schematic diagram of DMD process using a four-jet powder nozzle. (**B**) DMD process during additive manufacturing of a cylindric demonstration part.

Laser cladding experiments were performed using a diode laser (Laserline LDM 4000 60, Laserline GmbH, Mülheim-Kärlich, Germany) with a maximum output power of 4 kW, 900 to 980 nm wavelength, and a beam parameter product of 66 mm mrad. The laser radiation was delivered via an optical fibre with a core diameter of 600 µm to a robot-guided working head. A 72 mm collimation lens and 250 mm focusing lens generated a circular spot with a diameter of 2.1 mm on the surface of the specimen. For providing the powder, a feeder system (GTV PF2/2, GTV Verschleißschutz GmbH, Luckenbach, Germany) was used. Powder material was fed through a four-jet powder nozzle (Fraunhofer Coax12V7, Fraunhofer IWS, Dresden, Germany) with a circular powder spot diameter of 2–3 mm. As carrier and shielding gas, Argon 4.6 was used. The carrier gas flow was set to 5 L/min, and the shielding gas flow was set to 10 L/min. Figure 1A shows the schematic diagram of the DMD working head including the laser beam and powder jet orientation. Figure 1B shows the real DMD working head during additive manufacturing of a cylindric demonstrator. 

**Figure 2:**
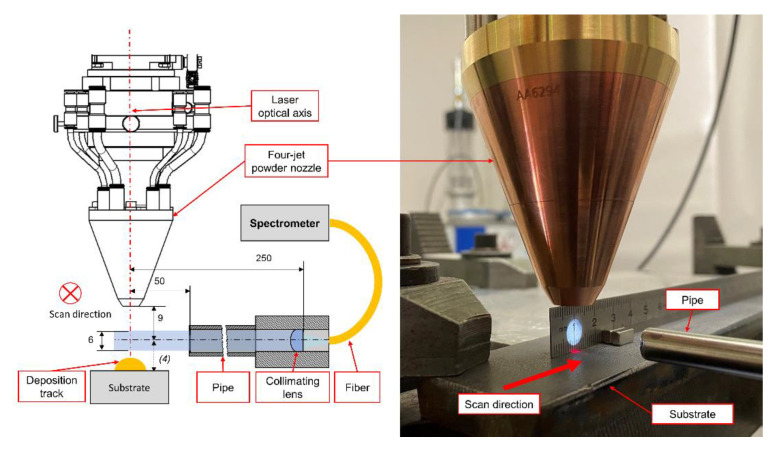
Measurement setup and collimation lens alignment.

Data acquisition was performed with the use of an UV-Vis spectrometer (OCEAN-HDX-UV-VIS, Ocean Insight, Orlando, FL, USA) with a spectral measurement range from 200 to 800 nm. The resolution at FWHM is 0.73 nm using a 10 µm entry slit. For efficient light collection, a fused silica collimation lens was attached horizontally to the processing head and aligned to the optical centre line of the laser beam. A pipe with 6 mm inner diameter and a blackened inner cylinder was attached to the collimation lens to reduce the impact of scattered radiation. The distance between the collimation lens and the optical axis of the laser was set to 250 mm. Cladding was performed at an orthogonal orientation of the collimation lens with respect to the processing direction. The lower edge of the collimation beam was set at a distance of 1 mm above the substrate surface. Figure 2 shows the measurement setup and collimation lens alignment. Coupling of the lens to the spectrometer was achieved by a multi-mode fibre with a core diameter of 100 µm.

**Figure 3:**
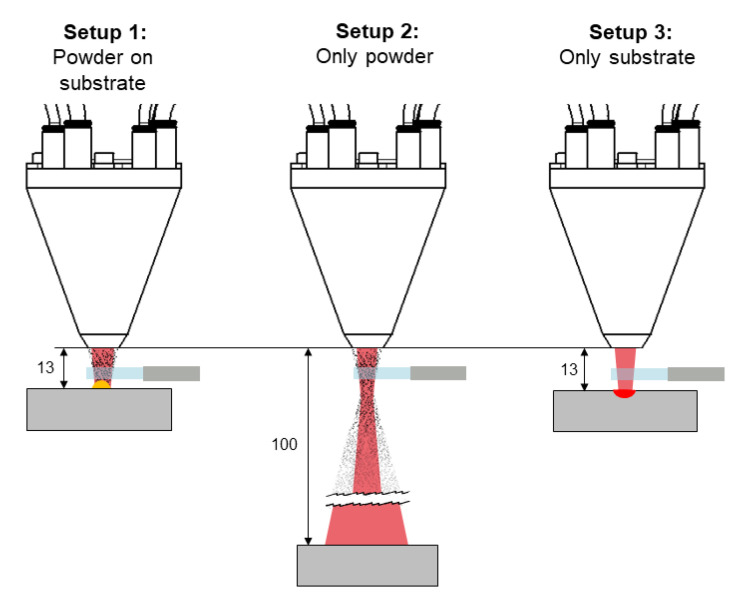
Experimental setups investigated in the present work.

Transient spectral measurements were performed using a constant integration time of 10 ms during the cladding process. Measurements were recorded and stored without any interruptions. The delay between the measurements averages 100 µs. According to the 10 ms integration time, the delay is negligible. In order to identify the origin of spectra emission, three different experimental setups were used. For each setup, the laser power was increased in five parameter sets. In order to generate a track with a sound quality in terms of dilution between the filler material and base material on the surface with the DMD process, more than one parameter has to be changed. Increasing the power leads to a stronger burn into the base material. Therefore, the scan velocity has to be increased in the same manner so that the placed energy per unit length is kept constant. In addition, the mass per unit length has to be equal in every experiment in order to keep the ratio of delivered energy by the laser and delivered mass by the powder feeder in the same order. Hence, the powder feed rate and the scan velocity were increased equally, as listed in Table 1. This methodology of changing various parameters at once can deliver a process window for a cladded layer with sound quality. For each setup, a linear track with a length of 300 mm was carried out. The particularly investigated experimental setups are shown in Figure 3 and include.

- Setup 1: Powder material on the substrate,
- Setup 2: Only powder material without the substrate, and
- Setup 3: No powder material on the substrate.

In setup 1, the powder nozzle had a working distance of 13 mm above the substrate surface and the powder material was fed according to the parameter sets listed in Table 1. In setup 2, the working distance was increased to 100 mm above the substrate surface, and the powder material was fed according to the given parameter sets. Thus, no track was placed, and the substrate surface was heated by the expanded laser beam. In setup 3, the powder feed rate was set to 0 g/min, but the carrying gas flow remained at 10 L/min and the working distance remained at 13 mm. Hence, merely the substrate material is remelted. The position of the collimation lens remained constant for all setups. For each combination of experimental setup and parameter set, one experiment was performed.

In the course of experimentation, plates (400 mm × 40 mm × 12 mm) made of steel (S235) were used as substrate. The chemical composition of this steel was determined by arc spark optical emission spectrometry performed by a stationary metal analyser (Spectrolab, SPECTRO Analytical Instruments GmbH, Kleve, Germany). The analysis configuration was set to low-alloyed steel. Analysis results are averaged after three separate measurements. For each measurement, the upper side of the substrate material was grinded with P80 SiC-paper and cleaned with denatured alcohol. The applied powder material was a cobalt-based alloy (MetcoClad21). Its chemical composition was determined by energy-dispersive X-ray spectroscopy (EDX) (Quantax energy-dispersive X-ray spectrometer with X-Flash 5010 detector, Bruker Corporation, Billerica, MA, USA). The energy resolution < 129 eV. Both results are shown in Table 2.

**Figure 4:**
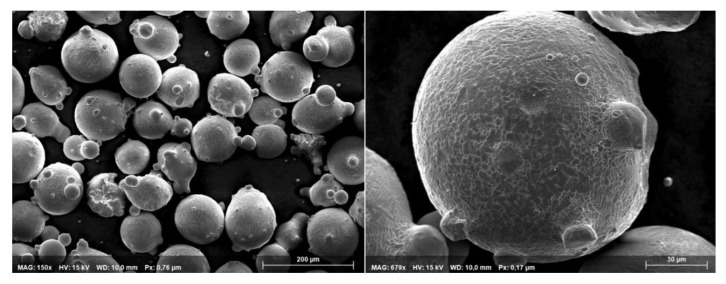
SEM imaging of powder particles MetcoClad21.

Additionally, Figure 4 shows the powder particles using a scanning electron microscope (SEM) (Phillips XL30 TMP, Philips Electron Optics, Eindhoven, The Netherlands). It can be seen that the particles are spherical in shape. Moreover, some smaller particles that are attached to bigger ones (satellites) have been found. From this SEM investigation, a sound flux ability and a stable continuous mass flow could be expected [32,33].

The spectra were measured back to back with a 10 ms integration time. The individual spectra were analysed and sorted in the first step. Single spectra were sorted into two types: In type A spectra, significant elemental peaks can be observed, whereas type B spectra feature no significant peaks. The identification of single peaks within type A spectra was carried out with the aid of the NIST Atomic Spectra Database [34]. Data are analysed for every experimental setup and parameter set. For the individual peak identification, a Matlab function called “findpeaks” has been used. The function also returns the peak intensity for type A spectra peaks. The peak intensity is described by the intensity of a peak minus the background radiation at this position. Figure 6 shows this definition at an exemplary type A spectrum. The chemical composition of both the substrate material and the powder material limit the quantity of possible emission lines to the following elements: C, Cr, Mn, Fe, Co, Mo, Cu, Si, Ni, Al, Ar, O, and N, where further possible traces and impurities are neglected. The corresponding element lines can be determined by transition probabilities and probable energy levels.

## 3. Results and Discussion

### 3.1. Deposition Track Analysis

**Figure 5:**
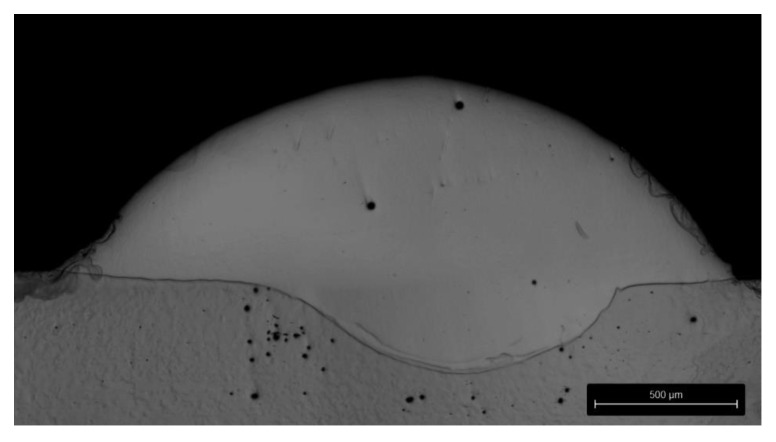
Cross-section cladded using experimental setup 1 and 2400 W laser power, 24 g/min powder feed rate, and 2 m/min scan velocity. Grinding with SiC-paper, polishing with 3 μm diamond suspension, and fine polishing with 0.05 μm colloidal silica.

Figure 5 shows a cross-sectional image of a single track cladded using experimental setup 1 and 2400 W laser power, 24 g/min powder feed rate, and 2 m/min scan velocity. Spherical pores with a maximum diameter of 25 µm have been detected within the deposition track. In relation to the dimensions of the track, these pores are negligible and indicate a qualitative coating. Additionally, the track geometry remains constant throughout the whole length of the track, as also observed for the other applied parameters.

### 3.2. Element Identification

**Figure 6:**
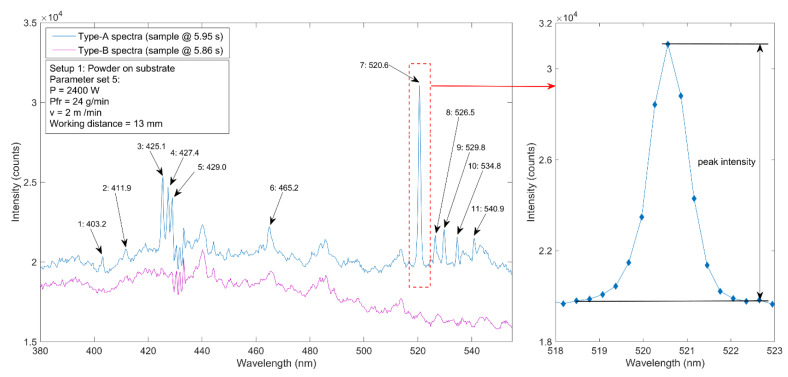
Identification of element lines via the evaluation of a single spectrum taken under the experimental conditions of setup 1 and parameter set 5 as defined in Figure 3 and Table 1.

Even though a larger wavelength range from 200 to 800 nm was considered during spectroscopic measurements, the most significant peaks were observed in a comparatively small range from 400 to 540 nm. Moreover, such peaks were not detected permanently in every single spectrum that was taken (compare definition of spectra types A and B in Section 2). An example of a well-evaluable spectrum of type A including the identification of significant spectral lines is shown in Figure 6. A type B spectrum is shown as well.

The relatively broad line at an observation wavelength of 403.2 nm most likely follows from the superposition of the two Mn I transitions at 403.076 nm and 403.307 nm; see Table 3. Such superposition of single spectral lines, namely the Co I transitions at 411.877 nm and 412.132 nm, likely results in the broad line detected at 411.9 nm. Both elements, cobalt and manganese, are constituents in the used powder as verified by the results of the preliminary EDX measurements listed in Table 2. However, it should be noted that an accurate identification or separation of single Mn and Co element lines was not possible due to the comparatively low spectral resolution of the used spectrometer. The further significant peaks observed can mainly be attributed to another major constituent of the used powder, chromium. The three single lines measured at 425.1 nm, 427.4 nm, and 429.0 nm correspond to the chromium resonance triplet [35,36]. The Cr lines at higher wavelengths feature quite different shapes and appearances; both nearly symmetric single lines (e.g., at 529.8 nm) and rather broad and asymmetric lines (465.2 nm) are observed. In some cases, the detected peaks probably consist of several elemental lines, either including other elements—that are not considered in the present work—or single Cr lines. The latter effect might apply to the moderately broad line at 520.6 nm. It is most likely composed of the three closely neighboured Cr I transitions at 520.44981 nm, 520.60229 nm, and 520.84094 nm. The first two of these lines are known to be asymmetric [34], explaining the slight asymmetry of the observed line. The most significant lines observed in Figure 6 are listed in Table 3.

It turns out that the detected peaks are provided by non-ionised but excited atoms (Mn I, Co I, and Cr I). No peaks of ionised metals (Mn II, Co II, and Cr II) could be detected in the region below 400 nm, even though the transmission of UV radiation is possible. Due to lower laser power, the DMD process is below the deep penetration threshold, which abruptly increases the penetration depth of the weld seam. This corresponds to increasing absorption of radiation caused by multiple reflections within evaporated material [40]. The cross-section shown in Figure 5 also indicates a heat-conduction process due to a low penetration depth of the weld seam. Moreover, the observed and detected lines within the measured spectra originate from transitions in the lower energy level range. This implies that preferentially, the incident laser irradiation causes the excitation of moderate electronic states within the powder material instead of an ionisation of its elements. This fact can be explained by the low applied laser power of a maximum of 2.4 kW (see Table 1). For instance, the first ionisation energy of chromium amounts to 653 kJ/mol. Thus, the incident laser intensity is most likely not sufficient for ionisation. However, it can be summarised that the major metallic constituents of the powder as detected via EDX are also found and quite clearly defined in the measured type A spectra.

### 3.3. Dependency of Line Intensity on Laser Power

**Figure 7:**
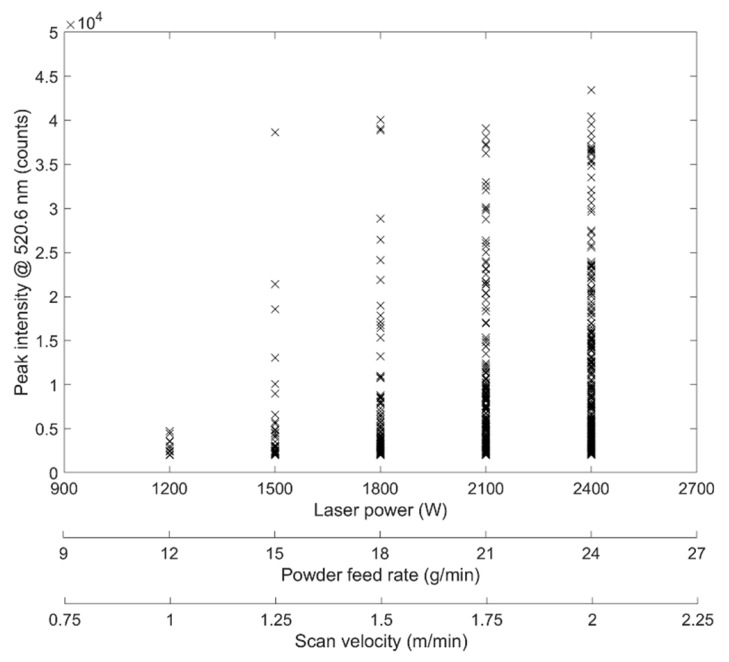
Peak intensity for Cr I line @ 520.6 nm vs. laser power, powder feed rate, and scan velocity, respectively. Data points are generated from all type A spectra of a single deposition track.

In order to gain information on the impact of applied laser process parameters on the formation and radiance of spectra, the intensities of a selected peak—the most intense one at a wavelength of 520.6 nm—was plotted as a function of laser power, powder feed rate, and scan velocity, respectively. This peak was chosen since it is composed of three different Cr I transitions as mentioned above and listed in Table 3. These transitions feature a very high intensity [34]; thus, the signal is prominent and easy to detect. In Figure 7, the peak intensities for each type A spectra are shown. 

It can be seen that the values for peak intensities increase with increasing laser power. In addition, the number of measurable peaks is also increasing. Such behaviour is also quite typical for laser-induced heating where the surface temperature of irradiated metal increases linearly with increasing incident laser power [41,42]. Thus, it can be assumed that the observed increase in peak intensity is—to a certain extent—simply caused by the temperature rise of the irradiated powder material. This presumption is also supported by the fact that with increasing laser power, an increase in background was observed in the particular spectra. As this background showed the basic shape of blackbody radiation, the impact of thermal processes becomes verisimilar.

### 3.4. Spectrum Type Distribution

**Figure 8:**
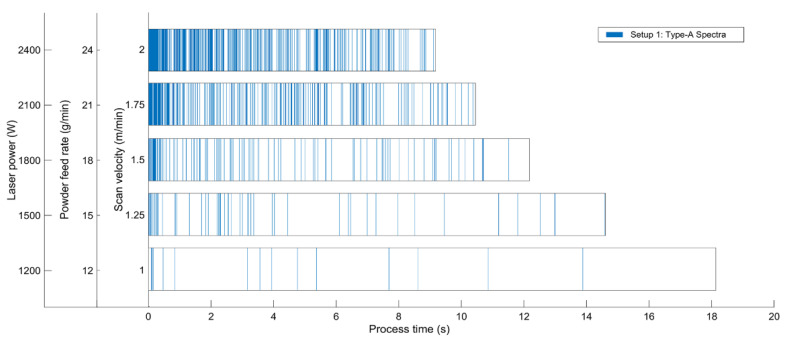
Incidence of detected type A spectra for setup 1 vs. process time; each blue line represents a type A spectrum whereas for the white ranges, merely type B spectra occurred. Note that the process time decreases with increasing scan velocity, since the process parameters were adapted to obtain a constant linear energy and linear mass as described in Section 2.

As already mentioned above, evaluable spectra of type A with distinct and characteristic lines or peaks were not obtained throughout the experiment. Thus, the incidence of such spectra as a function of process time and parameters was evaluated, as shown in Figure 8. This consideration was performed for spectra taken for all parameter sets in setup 1.

It turns out that the occurrence and incidence of type A spectra increases with increasing laser power. Moreover, the incidence is much higher at the beginning of the experiments, i.e., for shorter process times. With increasing process time, the density of type A spectra decreases notably. For the very beginning of the laser process, the high number of such spectra can be explained by an excessively high surface heating, since at the cut-in point of the incident laser irradiation, the scan velocity amounts to 0 m/min for a certain duration in the range of several milliseconds. This leads to a higher energy input and thus a local accumulation of heat and thermal glow, respectively. The highest type A spectra density is found for the highest laser power at the beginning of the process where such thermal influence has the most significant impact. This observation is in good accordance with the assumption stated in Section 3.3: The higher the temperature and incident photon density, the more evaluable spectra are formed. 

**Figure 9:**
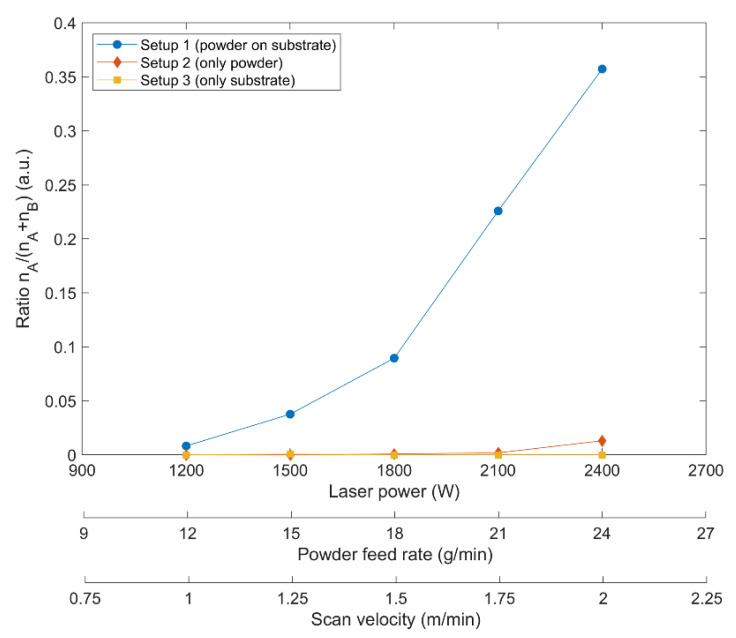
Incidence of detected type A spectra for setups 1–3 vs. laser power, powder feed rate, and scan velocity, respectively.

This assumption is also supported when comparing the particular ratio of type A spectra for the three investigated experimental settings visualised in Figure 3. This ratio, given by the quotient of the number of type A spectra n_A_ with respect to the total number of spectra n_A_ + n_B_ and thus the incidence of detected type A spectra, drastically increases with increasing laser power, powder feed rate, and scan velocity in the case of setup 1, as shown in Figure 9.

In contrast, marginal changes were observed for setup 2 where type A spectra were detected very rarely and only for higher laser powers. For setup 3, no type A spectrum was recorded. This observation can be explained by the experimental conditions during data acquisition. Setup 1 is the most suitable for heat accumulation within the considered volume of the power since here, both the heat emission of the substrate and the heated powder are observed. In setup 2, merely the directly laser-heated powder is detected, leading to a lower temperature and emission of spectra, respectively. This also applies to setup 3, where merely heat emission from the substrate surface was measured.

## 4. Conclusions

Laser cladding experiments with five different sets of parameters have been performed. The process emission has been inspected with a spectrometer in the process. Moreover, the emission of the laser powder interaction itself and the emission of the laser and base material interaction have been measured. It turned out that discrete line spectra only occurred in the process containing powder jet and base material. The spectral emission of the process was changing over time and correlated to the set of parameters. In conclusion, the following key facts have been shown:

- Spectral element lines have been observed in the laser cladding process.
- Element lines are provided by non-ionised atoms. Lines of ionised atoms have not been found. Cr lines have been observed more frequently compared to Co and Mn lines.
- With increasing laser power, the incidence and peak intensity of element lines increase.
- The observed correlation between the laser power, the powder feed rate, and the scan velocity on the one hand and the line intensity on the other hand opens an interesting aspect: The measurement of a line or spectrum intensity could be used for dynamic process control. A first analysis of cross-sections (compare Figure 5) revealed a certain dependency of the cladding dilution and the type of observed spectrum. Extensive investigations on this aspect will be carried out in ongoing work. Moreover, the footprint of full spectra may be suitable for an indirect in-line detection or monitoring of the composition of the laser-molten powder material. This approach seems to be promising for avoiding the deposition of non-stoichiometric layers.

Overall, it could have been shown that the spectral lines occur only rarely, even though based on the SEM analysis of the powder material, a constant and stable powder flux is most likely. The higher the laser power, the more often spectral lines occur in the process. This is valid only when the powder jet is switched on. The characterisation of the mechanisms that lead to the emission of discrete spectral lines rather than a continuous thermal spectrum only frequently is investigated in ongoing work.

## Figures and Tables

**Table 1:** Process parameter sets.

Parameter Set	Setup	Laser Power (W)	Scan Velocity (m/min)	Powder Feed Rate (g/min)	Working Distance (mm)
1	1	1200	1	12	13
	2				100
	3			0	13
2	1	1500	1.25	15	13
	2				100
	3			0	13
3	1	1800	1.5	18	13
	2				100
	3			0	13
4	1	2100	1.75	21	13
	2				100
	3			0	13
5	1	2400	2	24	13
	2				100
	3			0	13

**Table 2:** Chemical composition of substrate material (S235) measured via optical emission spectrometry and powder material (MetcoClad21) measured via EDX.

Material	C	Si	Mn	Cr	Mo	Ni	Al	Co	Cu	Fe
(wt.%)										
Substrate (S235)	0.10	0.19	1.02	0.03	0.01	0.03	0.03	0.01	0.02	98.56
Powder (MetcoClad21)	3.49	0.88	0.74	25.21	4.93	3.10	-	61.65	-	-

**Table 3:** Observed peaks as well as corresponding element lines including the corresponding energy levels. Data taken from [34].

Peak No.	Observed Wavelength (nm)	Element Line (nm)	$\mathrm{Energy}\;\mathrm{Levels}\;E_{i}-E_{k}\;\;\left(cm^{-1}\right)$	Line Ref.
1	403.2	403.076 (Mn I)	0–24,802.25	[37]
		403.307 (Mn I)	0–24,788.05	
2	411.9	411.877 (Co I)	8460.81–32,733.07	
		412.132 (Co I)	7442.41–31,699.69	
3	425.1	425.43517 (Cr I)	0–23,498.8156	[38]
4	427.4	427.48117 (Cr I)	0–23,386.3419	
5	429.0	428.97307 (Cr I)	0–23,305.0026	
6	465.2	465.1291 (Cr I)	7927.441–29,420.8645	[39]
7	520.6	520.44981 (Cr I)	7593.1484–26,801.9009	[38]
		520.60229 (Cr I)	7593.1484–26,796.2691	
		520.84094 (Cr I)	7593.1484–26,787.464	
8	526.5	526.4153 (Cr I)	7810.7795–26,801.9009	
		526.57143 (Cr I)	7810.7795–26,796.2691	
9	529.8	529.66905 (Cr I)	7927.441–26,801.9009	
		529.82715 (Cr I)	7927.4–26,796.3	
		530.07451 (Cr I)	7927.4–26,787.5	
10	534.8	534.57959 (Cr I)	8095.2–26,796.3	
		534.83141 (Cr I)	8095.2–26,787.5	
11	540.9	540.97834 (Cr I)	8307.6–26,787.5	

## Data Availability

The data is available within the article and can be requested from the corresponding author.
